# Associations of ADL and IADL disability with physical and mental dimensions of quality of life in people aged 75 years and older

**DOI:** 10.7717/peerj.5425

**Published:** 2018-08-09

**Authors:** Robbert J. Gobbens

**Affiliations:** Faculty of Health, Sports and Social Work, Inholland University of Applied Sciences, Amsterdam, The Netherlands; Zonnehuisgroep Amstelland, Amstelveen, The Netherlands; Department of Primary and Interdisciplinary Care, Faculty of Medicine and Health Sciences, University of Antwerp, Antwerp, Belgium

**Keywords:** Older people, Disability, Quality of life

## Abstract

**Background:**

Quality of life is an important health outcome for older persons. It predicts the adverse outcomes of institutionalization and premature death. The aim of this cross-sectional study was to determine the influence of both disability in activities of daily living (ADL) and instrumental activities of daily living (IADL) on physical and mental dimensions of quality of life.

**Methods:**

A total of 377 Dutch people aged 75 years and older completed a web-based questionnaire. This questionnaire contained the Groningen Activity Restriction Scale (GARS) for measuring ADL and IADL and the Short-Form Health Survey (SF-12) for measuring quality of life. The SF-12 distinguishes two dimensions of quality of life, a physical and mental dimension.

**Results:**

All ADL disability items combined and all IADL disability items combined explained a significant part of the variance of the physical and the mental dimension of quality of life. Only ADL item “stand up from sitting in a chair”, and IADL items “do “heavy” household activities” and “do the shopping” were negatively associated with both quality of life dimensions after controlling for all the variables in the model.

**Discussion:**

This study showed that disability in ADL and IADL is negatively associated with quality of life in older people. Therefore, it is important for health care professionals to carry out interventions aimed to prevent and diminish disability or the adverse outcomes of disability such as a lower quality of life. In order to be effective these interventions should be inexpensive, feasible, and easy to implement.

## Introduction

Quality of life has been defined by the World Health Organization Quality of Life Group as “an individual’s perception of their position in life in the context of the culture and value system in which they live and in relation to their goals, expectations, standards and concerns” (World Health Organization Quality of Life Group, 1995, p. 1405). Quality of life in community-dwelling older people predicts the adverse outcomes of institutionalization and premature death, even after controlling for disability and frailty (Bilotta et al., 2011). To support independent living in older people, both health care and social care professionals may need to carry out preventive interventions focused on aspects related to quality of life, with the aim of delaying institutionalization and avoiding premature death. Determining the influence of disability on quality of life in older people is important to developing early detection of problems and conducting preventive interventions.

In addition to lower quality of life, disability is a relevant health outcome for older persons. There are several ways of defining disability. The most widely used is: experiencing difficulty in carrying out activities that are essential to independent living - difficulties in performing activities of daily living (ADL), and/or instrumental activities of daily living (IADL) (Tas et al., 2007a; Tas et al., 2007b). ADL functions are essential for an individual’s self- care (e.g., wash and dry your whole body and get on and off the toilet), whereas IADL functions are more concerned with self-reliant functioning in a given environment (e.g., prepare dinner and do the shopping). ADL disability represents a more severe and later form of disability than IADL disability (Hardy et al., 2005; Wong et al., 2010), resulting in a lower proportion of persons with ADL disability than IADL disability (Akosile et al., 2018; Chatterji et al., 2015).

Disability is associated with increased health care utilization and related costs (Fried et al., 2004), and premature death (Manton, 1988; Mor et al., 1994; Walter et al., 2001). In addition, disability is associated with impaired quality of life in older people (Akosile et al., 2018; Den Ouden et al., 2013; Gureje et al., 2006; Soósová, 2016). However, the “disability paradox” (Albrecht & Devlieger, 1999) suggests that persons with severe impairments may nevertheless report high quality of life (Watson, 2002), although this paradox seems to dissolve when contextual factors (i.e., personal and environmental situation) are considered (Fellinghauer et al., 2012). Disability has a dynamic nature, so persons can move in and out of disability, with transitions between states of disability (Hardy et al., 2005; Nikolova et al., 2011; Van Houwelingen et al., 2014). Transitions to greater disability were more common than improvements in disability in people aged ≥85 years (Van Houwelingen et al., 2014). In particular, people with more than one chronic disease, depressive symptoms, and cognitive impairment had the highest risk of deteriorating; however, a small number of very old people are able to improve in their disability status (Van Houwelingen et al., 2014).

The aim of the present cross-sectional study was to determine the influence of both ADL and IADL disability on quality of life, incorporating a physical and a mental dimension, in people aged 75 years and older. In contrast to previous research (Akosile et al., 2018; Den Ouden et al., 2013; Gureje et al., 2006; Soósová, 2016) the main focus here is on the associations between ADL and IADL items and quality of life. Items are more concrete than the types of disability (ADL, IADL) and thereby provide health care professionals (e.g., nurses, general practitioners, physiotherapists) and professionals working in the social domain (e.g., social workers, domestic help) specific targets to enhance quality of life in older people. To enhance quality of life of older people, it is relevant to know which items of ADL and IADL are associated with lower quality of life because the interventions will be carried out by different professionals. For example, if an older person can no longer wash and undress themselves, a district nurse can provide support (e.g., in the Netherlands) and if an older person has difficulties performing household activities then domestic help can provide the necessary support.

## Methods

### Study population and data collection

The Senioren Barometer is a web-based questionnaire used to assess the opinion of a panel of Dutch older people (aged 50 years and older) about different aspects of life. This questionnaire has been used in previous studies (Gobbens, Luijkx & Van Assen, 2013; Gobbens, Van Assen & Schalk, 2014).

In the period from December 2009 to January 2010 1,492 respondents completed at least part of the questionnaire, of whom 1,031 filled out the section on background characteristics, quality of life, and disability. Because disability is associated with greater age (Tas et al., 2007a; Tas et al., 2007b) the author selected only people aged 75 years and older (*n* = 377). As described in previous studies using the Senioren Barometer, older people can volunteer, and participation is always without obligation. The sample was invited to participate in the study in different ways and through multiple sources. First, people could indicate through the website (http://www.seniorenbarometer.nl) that they wanted to complete the questionnaire. Second, organizations for older people in the Netherlands were approached and asked to issue an announcement of the study on their websites so that their members who were interested could register. Third, a major source of participants was persons who attended computer training courses for older persons given by a large training and educational institute in the Netherlands.

Medical ethics approval was not necessary as particular treatments or interventions were not offered or withheld from respondents. The integrity of respondents was not encroached upon as a consequence of participating in the study, which is the main criterion in medical-ethical procedures in the Netherlands (Central Committee on Research inv. Human Subjects, 2010). Informed consent in relation to detailing the study and maintaining confidentiality was observed.

### Measures

#### Quality of life

The author used the Short-Form Health Survey (SF-12) for measuring quality of life (Ware Jr, Kosinski & Keller, 1996). The SF-12 is a shorter version of the SF-36 (Ware Jr & Sherbourne, 1992) that uses only 12 questions. The SF-12 is developed to replicate the SF-36 with the aim to minimalize respondent burden. The 12 items are used to derive two summary quality of life measures, the physical dimension (six items) and the mental dimension (six items); their scores range from 0 to 100. Higher scores refer to better quality of life. Several studies have reported the validity and reliability of the SF-12 as a measure of quality of life in the general population, including older people (Bentur & King, 2010; Cernin et al., 2010; Jakobsson et al., 2012; Kim et al., 2014; Kontodimopoulos et al., 2007). In the present study, the (unstandardized) Cronbach’s alpha was .82 for the physical dimension and .73 for the mental dimension; an adequate value of the Cronbach’s alpha is between .70 and .90 (Cortina, 1993).

#### Disability

The author used the Groningen Activity Restriction Scale (GARS) for assessing disability (Kempen & Suurmeijer, 1990). The GARS is a self-report questionnaire consisting of two subscales. The first subscale measures ADL (11 items) and the second subscale relates to IADL (seven items). Each item has four response options: (1) able to perform the activity without any difficulty, (2) able to perform the activity with some difficulty, (3) able to perform the activity with great difficulty, and (4) unable to perform the activity independently. A distinction can then be made in two categories, complete independence and dependency (more or less). The disability total score ranges from 18 (no disability) to 72 (maximum disability). Following Ormel et al. (2002) the cut-point of 29 has been chosen for the disabled group because this cut-point corresponds with the 85th percentile of the GARS in a large sample of older people (Kempen et al., 1996b). The scores for the ADL and IADL subscales range from 11 to 44 and 7 to 28, respectively, with higher scores indicating greater disability; cut-points for these subscales do not exist. The GARS has shown good psychometric properties for assessing disability in older people (Kempen et al., 1996a). In this study, the (unstandardized) Cronbach’s alpha’s for ADL and IADL disability were .82 and .80, respectively, representing adequate values (Cortina, 1993)

#### Background characteristics: sociodemographic and multimorbidity

The sociodemographic background characteristics considered were age, sex, marital status, education level, and net household income. See Table 1 for a detailed description of the answer categories. Multimorbidity was assessed by asking the respondents, “Do you have two or more diseases and/or chronic disorders?” (yes/no).

### Analysis strategies

First, the author determined the characteristics of the sample using descriptive statistics. Second, the quality of life dimensions (physical, mental) scores for non-disabled and disabled participants were compared using student’s *t*–tests assuming unequal population variances. Effect size was assessed with Cohen’s *d*, assuming equal population variances; .2, .5, .8 corresponding to small, medium, large effect size, respectively (Cohen, 1988). Correlations of ADL and IADL disability with the physical and the mental dimensions of the SF-12 were also examined. According to Cohen, correlations were considered as small, medium, or large with coefficients of .1, .3, or .5, respectively (Cohen, 1988).

**Table 1:** Participant characteristics (*N* = 377).

Characteristic	*n*(%)
Age, mean ± SD, range	79.8 ± 3.7, 75–95
Sex, % of men	261 (69.2)
Marital status	
Married or cohabiting	244 (64.8)
Single	36 (9.5)
Divorced	11 (2.9)
Living apart together	3 (0.8)
Widowed	83 (22.0)
Education	
None	30 (8.0)
Primary	34 (9.0)
Secondary	160 (42.4)
Polytechnics and higher vocational training	113 (30.0)
University	40 (10.6)
Income^a^	
€999 - or less	7 (2.1)
€1,000–€1,499	44 (13.2)
€1,500-€1,999	54 (16.1)
€2,000–€2,499	90 (27.0)
€2,500–€2,999,-	54 (16.1)
€3,000–€3,499,-	38 (11.4)
€3,500–€3,999,-	25 (7.5)
€4,000–€4,499,-	11 (3.3)
€5,000 or more	11 (3.3)
Multimorbidity, % yes	166 (44.0)
GARS	
Total disability	95 (25.2)
ADL disability, mean ± SD, range	13.6 ± 3.8,11–33
Dress yourself	55 (14.6)
Get in and out of bed	31 (8.2)
Stand up from sitting in a chair	53 (14.1)
Wash your face and hands	6 (1.6)
Wash and dry your whole body	57 (15.1)
Get on and off the toilet	12 (3.2)
Feed yourself	4 (1.1)
Get around in the house (if necessary, with a cane)	18 (4.8)
Go up and down the stairs	134 (35.5)
Walk outdoors (if necessary, with a cane)	75 (19.9)
Take care of your feet and toenails	183 (48.5)
IADL disability, mean ± SD, range	11.2 ± 4.5, 7–28
Prepare breakfast or lunch	14 (3.7)
Prepare dinner	88 (23.3)
Do “light” household activities	69 (18.3)
Do “heavy” household activities	212 (56.2)
Wash and iron your clothes	169 (44.8)
Make the beds	185 (49.1)
Do the shopping	86 (22.8)
SF-12	
Physical dimension of quality of life, mean ± SD, range	66.9 ± 25.6, 0–100
Mental dimension of quality of life, mean ± SD, range	74.5 ± 18.7, 10–100


**Notes.**

a 43 missing values (11.4%).

SD Standard deviation GARS Groningen Activity Restriction Scale ADL Activities of Daily Living IADL Instrumental Activities of Daily Living SF-12 Short-Form Health Survey - 12

Before carrying out regression analyses some sociodemographic variables were coded for analysis. As in a previous study, the author created dummies for sex (“1” woman, “0” man), marital status (“1” married or cohabiting, “0” rest) and multimorbidity (“1” yes, “0” no), and linear effects of age and level of education were incorporated into the analyses (Gobbens, Luijkx & Van Assen, 2013). Bivariate associations between one background variable or disability item on the one hand and one quality of life dimension (physical, mental) on the other hand were tested using regression analyses. Subsequently, the author examined the effects of each variable (background variables, disability items) on the physical and mental dimensions in four multiple linear regression analyses, controlling for all the other variables in the model. The simplest model only assessed the effects of all background variables together. One model also included all 11 ADL disability items, whereas another model also included the seven IADL items together with the background variables. The most complex model included all 24 items. The fit (explained variance) of all four models was tested (*R*^2^) and compared (delta *R*^2^). Power analyses using GPower 3.1.0 (Faul et al., 2007) showed that the sequential linear regression analyses on 377 participants had a power of at least 80% to detect an effect of Cohen’s *f*^2^ = .056 which is a small to medium effect size (Cohen, 1988).

Data were processed using SPSS version 24.0 (IBM Corporation, Armonk, NY, USA). All reported *p*-values are two-tailed. A *p*-value <0.05 was considered statistically significant.

## Results

### Participant characteristics

See Table 1 for an overview of the descriptive statistics of the participant characteristics. The mean age of the participants was 79.8 (SD = 3.7), 69.2% were male, and 64.8% were married or cohabiting. The average scores on quality of life for the physical and mental dimensions were 66.9 (SD 25.6) and 74.5 (SD 18.7), respectively. Using the cut-point of 29 on the GARS, 25.2% of the participants were totally disabled, including both the ADL and the IADL subscale. In addition, 54.6% and 67.4% of the participants had at least one ADL disability and IADL disability, respectively. Of the 11 ADL disability items, participants experienced the greatest dependency in relation to taking care of their feet and toenails (48.5%). Of the 7 IADL disability items, participants experienced the greatest dependency in relation to doing “heavy” household activities (56.2%). In general, it should be noted that the percentages of the IADL disability items are higher than the percentages of the ADL disability items; five IADL disability items scored higher than 20% versus two ADL disability items (see Table 1).

### Differences between non-disabled and disabled participants on quality of life

Table 2 presents the results of comparing disabled and non-disabled people on the physical and the mental dimensions of the SF-12. Disabled participants scored lower on both quality of life dimensions (*p*-values < 0.001), with very large effect sizes, *d* = 1.30 for the mental dimension and *d* = 1.82 for the physical dimension.

**Table 2:** Comparison of quality of life dimensions between disabled and non-disabled participants.

	Non-disabled *n* = 279 M (SD)	Disabled *n* = 95 M (SD)	Results *t*-test^a^	Effect size Cohen’s d^b^
Physical dimension of quality of life	76.19 (19.29)	39.61 (22.27)	*t*(144.95) = 14.29 < 0.001	*d* = 1.82
Mental dimension of quality of life	79.85 (14.59)	58.68 (20.46)	*t*(128.05) = 9.31 < 0.001	*d* = 1.30

**Notes.**

a Assuming unequal population variances.

b Assuming equal population variances.

### Correlations between disability and quality of life

Table 3 shows the correlations between ADL disability, IADL disability, physical quality of life, and mental quality of life. Most correlations were strong (>5); only the correlation between ADL disability and mental quality of life could be considered as medium (.483) (all *p*-values < 0.001).

**Table 3:** Correlations between ADL disability, IADL disability, physical and mental dimensions of quality of life.

	IADL disability	Physical quality of life	Mental quality of life
ADL disability	0.702	−0.683	−0.483
IADL disability		−0.676	−0.541
Physical quality of life			0.734

**Notes.**

All correlations were significant at *p* <.001.

### Regression analyses: effects of ADL and IADL disability items on quality of life

Table 4 presents the results of the bivariate and sequential linear regression analyses on the physical and mental quality of life dimensions of the SF-12. The table shows the effects of six background characteristics, 11 ADL disability items, and seven IADL items on the two dimensions of quality of life (physical, mental). Columns 2–4 and 8–10 present the bivariate regressions. Being a man, younger age, married or cohabiting, higher education, higher income, and no multimorbidity were associated with higher scores on both the physical and mental dimensions. Of the 11 ADL disability items, all were associated with physical quality of life and 10 were associated with mental quality of life. The exception was the item “feed yourself” (*p* = 0.058). All seven IADL disability items were associated with both quality of life dimensions.

**Table 4:** Effect of background characteristics, ADL and IADL disability items on the physical and mental dimensions of quality of life.

	Physical dimension of quality of life						Mental dimension of quality of life					
	Bivariate			Multiple			Bivariate			Multiple		
	B	SE	*p*	B	SE	*p*	B	SE	*p*	B	SE	*p*
*Background characteristics*												
Sex (women)	−11.92	2.81	**<0.001**	−2.81	2.15	0.192	−4.89	2.08	**0.019**	2.26	2.10	0.283
Age	−0.73	0.36	**0.040**	0.27	0.23	0.251	−0.81	0.26	**0.002**	−0.06	0.23	0.801
Marital status (married)	8.48	2.74	**0.002**	−0.83	2.08	0.692	5.18	2.01	**0.010**	0.99	2.04	0.626
Education	4.86	1.26	**<0.001**	0.62	0.88	0.481	2.86	0.92	**0.002**	0.22	0.86	0.795
Income	3.60	0.74	**<0.001**	0.33	0.52	0.524	1.92	0.52	**<0.001**	0.35	0.51	0.496
Multimorbidity	−29.13	2.21	**<0.001**	−13.35	1.82	**<0.001**	−13.50	1.82	**<0.001**	−4.03	1.78	**0.024**
Δ*R*^2^				0.364		**<0.001**				0.162		**<0.001**
*ADL disability items*												
Dress yourself	−27.20	2.45	**<0.001**	−6.98	3.09	**0.024**	−12.63	1.96	**<0.001**	−0.12	3.02	0.967
Get in and out of bed	−32.04	3.72	**<0.001**	−6.95	3.42	**0.043**	−17.72	2.82	**<0.001**	−6.57	3.34	0.050
Stand up from sitting in a chair	26.94	2.90	**<0.001**	−5.68	2.59	**0.029**	−16.63	2.19	**<0.001**	−5.55	2.53	**0.029**
Wash your face and hands	−27.89	5.92	**<0.001**	2.07	5.50	0.707	−12.96	4.39	**0.003**	2.70	5.37	0.615
Wash and dry your whole body	−24.35	2.19	**<0.001**	2.01	3.10	0.516	−12.28	1.73	**<0.001**	0.95	3.03	0.754
Get on and off the toilet	−37.37	7.27	**<0.001**	0.99	4.94	0.841	−25.07	5.33	**<0.001**	−4.28	4.82	0.375
Feed yourself	−40.77	12.72	**0.001**	−11.28	9.22	0.222	−17.79	9.36	0.058	−3.12	9.00	0.729
Get around in the house (if necessary, with a cane)	−24.78	4.24	**<0.001**	6.43	3.37	0.057	−13.93	3.15	**<0.001**	1.33	3.29	0.686
Go up and down the stairs	−21.00	1.23	**<0.001**	−5.78	1.63	**<0.001**	−10.62	1.07	**<0.001**	−0.84	1.59	0.597
Walk outdoors (if necessary, with a cane)	−20.33	1.60	**<0.001**	0.03	1.82	0.985	−11.03	1.28	**<0.001**	0.93	1.77	0.601
Take care of your feet and toenails	−10.80	0.88	**<0.001**	1.41	0.86	0.101	−5.29	0.71	**<0.001**	1.35	0.83	0.108
Δ*R*^2^				0.058		**<0.001**				0.045		**0.012**
*IADL disability items*												
Prepare breakfast or lunch	−14.34	4.11	**0.001**	6.83	3.34	**0.042**	−7.97	3.01	**0.009**	2.07	3.26	0.526
Prepare dinner	−5.89	1.42	**<0.001**	0.74	1.15	0.517	−3.29	1.05	**0.002**	0.003	1.12	0.998
Do “light” household activities	−19.29	1.74	**<0.001**	−1.52	1.68	0.368	−9.83	1.37	**<0.001**	0.83	1.64	0.613
Do “heavy” household activities	−14.27	0.75	**<0.001**	−6.57	1.05	**<0.001**	−8.64	0.62	**<0.001**	−4.55	1.03	**<0.001**
Wash and iron your clothes	−7.49	1.05	**<0.001**	−1.03	0.91	0.260	−4.03	0.79	**<0.001**	0.58	0.89	0.516
Make the beds	−13.05	0.88	**<0.001**	−1.23	1.07	0.254	−7.90	0.70	**<0.001**	−1.83	1.05	0.083
Do the shopping	−18.06	1.37	**<0.001**	−5.41	1.42	**<0.001**	−11.13	1.06	**<0.001**	−5.74	1.39	**<0.001**
Δ*R*^2^				0.108		**<0.001**				0.135		**<0.001**
Δ*R*^2^ ADL and IADL				0.350		**<0.001**				0.282		**<0.001**
*R*^2^ total				0.714		**<0.001**				0.444		**<0.001**

**Notes.**

All *p*-values significant at 0.05 are printed in bold.

Columns 5–7 and 11–13 summarize the results of the sequential linear regression analyses. *R*^2^ total indicates that 71.4% and 44.4% of the physical and mental quality of life dimensions were explained by all the predictors together, respectively. After controlling for the background variables (sociodemographic characteristics, multimorbidity), disability (ADL and IADL items together) explained 35.0% of physical quality of life and 28.2% of mental quality of life, with both *p*-values <0.001. The ADL disability items together explained 5.8% and 4.5% of the physical and mental dimension, with *p*-values <0.001 and 0.012, respectively, after controlling for all background characteristics and IADL disability items, representing a medium to large effect size (*f*^2^ = .20) and a small to medium effect size (*f*^2^ = .08), respectively. The IADL disability items together explained a significant part of both quality of life dimensions after controlling for background characteristics and ADL items, with increases in explained variance of 10.8% (physical; *f*^2^ = .38, large effect size) and 13.5% (mental; *f*^2^ = .24, medium to large effect size) (both *p*-values <  0.001).

In addition, Table 4 presents the effects of each of the background characteristics and individual ADL and IADL items on physical and mental quality of life. The columns five and 11 show the regression coefficients with corresponding standard errors (columns six and 12) and *p*-values (columns seven and 13).

Before interpreting the effects of individual items after controlling for the other variables, the author checked for multicollinearity. As the variance inflation factors (VIF) for all items were smaller than 5, which is below the threshold of 10 (Yu, Jiang & Land, 2015), the author relied on his estimates as they are not strongly affected by multicollinearity.

Of the background variables, only multimorbidity was negatively associated with quality of life, both physical and mental. None of the other background characteristics were associated with quality of life, after controlling for all the other variables in the model.

Of the 11 ADL disability items only four were significantly associated with quality of life. The ADL item “stand up from sitting in a chair” was negatively associated with both dimensions (physical, mental). The ADL items “dress yourself”, “get in and out of bed”, and “go up and down the stairs” were only negatively associated with the physical dimension of quality of life. Of the seven IADL disability items, three were associated with quality of life. The two IADL items (do “heavy” household activities, do the shopping) were negatively associated with both the physical and mental dimensions of quality of life and “prepare breakfast or lunch” was positively associated with the physical dimension. All effect sizes (*f*^2^) of the individual ADL and IADL disability items on physical as well as mental quality of life were <.15, representing small effect sizes. Of all ADL disability items, “go up and down the stairs” and “stand up from sitting in a chair” had the largest effect sizes on the physical and mental quality of life dimensions, *f*^2^ = .042 and *f*^2^ = .016, respectively. Of all IADL disability items, the item with the largest effect sizes on the physical as well as the mental dimension of quality of life was “do “heavy” household activities”, with *f*^2^ = .13 and .065, respectively.

## Discussion

In this study the author determined the associations between ADL and IADL disability items and quality of life in a sample consisting of 377 Dutch people aged 75 years or older. The author used two validated questionnaires, the GARS for assessing disability and the SF-12 for assessing quality of life, containing a physical and a mental dimension. To the best of my knowledge, the present study was the first using the GARS and the SF-12 to determine the associations between disability and quality of life. In addition, no previous study paid attention to the predictive value of the individual ADL and IADL disability items on quality of life.

The bivariate regression analyses showed that the following factors were associated with physical quality of life as well as mental quality of life: being a man, younger age, married or cohabiting, higher education, higher income, no multimorbidity, ten ADL disability items, and seven IADL disability items. The ADL disability item “feed yourself” was not associated with the mental dimension. However, the sequential linear regression analyses revealed that only multimorbidity, ADL item “stand up from sitting in a chair”, and IADL items “do ‘heavy’ household activities” and “do the shopping” were significantly associated with both quality of life dimensions, after controlling for all the variables in the model.

The finding that multimorbidity is associated with lower quality of life in older people is supported by previous studies in several countries using different measurement instruments (Brettschneider et al., 2013; Fortin et al., 2006; Garin et al., 2014; Gu et al., 2018). In Germany, quality of life of multimorbid people aged 65 to 85, assessed with the EQ-5D and the EQ-5D visual analogue scale (EQ-VAS) (Rabin & De Charro, 2001), decreased with an increasing count and severity of chronic conditions (Brettschneider et al., 2013). In Canada, 238 people completed the SF-36 (Ware Jr & Sherbourne, 1992) for assessing quality of life, and multimorbidity was measured by counting the number of chronic diseases and with the Cumulative Illness Rating Scale (CIRS) (Linn, Linn & Gurel, 1968); this study showed that the physical health dimension of quality of life deteriorated more than the mental health dimension of quality of life with increasing multimorbidity (Fortin et al., 2006). A study among Spanish people (≥50 years) also demonstrated that the number of chronic diseases was associated with lower quality of life (Garin et al., 2014), assessed with the WHOQOL-AGE (Caballero et al., 2013). Finally, a longitudinal study conducted in China showed that distinct multimorbidity patterns had various impacts on different dimensions of quality of life among community-dwelling older people (Gu et al., 2018). These findings are important because multimorbidity is frequently present in older people; in the age group 75–84 years the prevalence is 71.7% (Abad-Diez et al., 2014). The author recommends more studies focusing on the impact of multimorbidity patterns on quality of life in other countries. These studies should focus in particular on effects of combinations of common chronic diseases on quality of life, thereby providing direction to (preventive) interventions.

All ADL disability items combined explained a significant part of the variance of both the physical dimension and the mental dimension of quality of life. Another study showed that maintaining independence in ADL had a positive effect on four domains of the WHOQOL-OLD (sensory abilities; autonomy; past, present, and future activities; social participation) (Power, Quinn & Schmidt, 2005), and one domain of the WHOQOL-BREF (physical health) (The WHOQOL Group, 1998; Soósová, 2016)). Quality of life, assessed with the WHOQOL-OLD (Power, Quinn & Schmidt, 2005) and the WHOQOL-BREF (The WHOQOL Group, 1998), were significantly associated with ADL disability in two samples of Nigerian older people aged 65 years and older (Akosile et al., 2018; Gureje et al., 2006). A Dutch study including a total of 537 middle-aged and older persons also found that quality of life, assessed with the SF-36 (Ware Jr & Sherbourne, 1992), was associated with ADL disability, measured with the Katz-questionnaire (Katz & Akpom, 1976; Den Ouden et al., 2013). In particular, health care professionals (e.g., district nurses, physiotherapists, general practitioners, occupational therapists) should identify (potential) limitations in performing ADL at an early stage in order to maintain or increase quality of life in older people. Based on the present study, special attention is needed to address problems people have when standing from sitting, because this activity is associated with lower physical and mental quality of life.

All IADL disability items combined explained a larger part of the variance of both the physical and the mental dimension of quality of life compared with all ADL disability items together, 10.8% versus 5.8% and 13.5% versus 4.5%, respectively. Two studies referred to above also found that IADL disability was associated with quality of life (Akosile et al., 2018; Gureje et al., 2006). The finding that IADL disability items were more prevalent than ADL disability is supported by other studies (Akosile et al., 2018; Bleijenberg et al., 2017; Hu et al., 2012) and contributes to the evidence that IADL disability occurs earlier than ADL disability; probably because IADL is more complex and appeals more to cognitive function. In Nigeria the prevalence figure of IADL disability was 39.3% versus ADL disability 32.5% (Akosile et al., 2018). Among Dutch older people, with an average age of 74.6 years, carrying out household tasks was the most frequent problem (44.8%), followed by travelling (26.9%), and grocery shopping (23.0%) (Bleijenberg et al., 2017). In particular, the first and the last item are important because the present study showed that these two items were associated with the physical as well as the mental dimension of quality of life in older people. These findings have not been available to date. Conducting interventions on problems that older people can experience with performing heavy household activities and shopping could help them reach a higher quality of life. Domestic help may meet these needs or additionally reablement or restorative care services may be of benefit. These are short term services aimed at improving the independence of older people so they can hopefully go back to living independently without ongoing assistance.

The model including all the prediction variables explained a large part of the variance in scores of the physical and mental dimensions of the SF-12, 71.4% and 44.4%, respectively. In a sample of community-dwelling older Dutch people (*n* = 8,928) it was shown that people experiencing disability, multimorbidity, and frailty scored lower on quality of life compared with people experiencing individual conditions (Lutomski et al., 2014). It is possible that the explained variances in the scores of the quality of life dimensions were also greater if depression as a predictive variable was included in the model; a review, including 74 studies, found an association between depression and lower quality of life in older people, independent of how quality of life was assessed (Sivertsen et al., 2015).

This study has some limitations. First, the cross-sectional nature of this study does not allow strict cause–effect interpretations of the associations between the ADL and IADL disability items and quality of life. A longitudinal study is recommended to establish such associations. Second, disability was assessed by the GARS, a self-report measure, that does not include performance-based measures. A combination of both measures may be the best way to fully capture the picture of disability in ADL and IADL. However, in a sample of oldest old (≥80 years) it was demonstrated that self-assessments for disability in ADL and IADL reliably reflect direct assessment in performance (Bravell, Zarit & Johansson, 2011). Third, the author used the Senioren Barometer for data collection. This is a web-based questionnaire, so access to Internet was necessary for participating in the present study; this may have led to selection bias. In this context, it should be noted that in the study sample 69.2% were men, while in the Dutch population aged 75 years and older, only 37.9% are men, as established January 1, 2010 (Statistics Netherlands, 2017).

## Conclusions

In this study the author showed that disability in ADL and IADL is negatively associated with quality of life in older people. Therefore, it is important for health care professionals to carry out interventions aimed at preventing and diminishing disability or its adverse outcomes, such as a lower quality of life. Promising interventions are multidisciplinary and multifactorial in nature, should be preceded by an individualized assessment, and should involve case management and long-term follow up (Daniëls et al., 2010). Lifestyle interventions targeting physical exercise, nutrition, and cognition appear to be effective against disability in ADL and IADL; in order to be actually effective, these interventions should be inexpensive, feasible, and easy to implement (Fougère et al., 2018). In line with the findings of the present study, it is recommended to first focus on the disability items that have the greatest impact on quality of life of older people (“stand up from sitting in a chair”, “do ‘heavy’ household activities” and “do the shopping”) to achieve the best outcome.

##  Supplemental Information

10.7717/peerj.5425/supp-1 Supplemental Information 1 Raw data exported from the Seniorenbarometer 2009 applied for data analysis and preparation for Tables 1– 4

## Additional Information and Declarations

### Data Availability

The following information was supplied regarding data availability: The raw data are provided in a Supplemental File.
